# Exploration of factors affecting parent-infant closeness and separation in hospitals in Nepal – a qualitative study

**DOI:** 10.1186/s12884-026-09387-5

**Published:** 2026-06-02

**Authors:** Olivia Brunell, Anna Bergström, Rejina Gurung, Leif Eriksson

**Affiliations:** 1 Department of Women’s and Children’s Health, Uppsala University, Uppsala, Sweden; 2 WOMHER- Women’s Mental Health during the Reproductive Lifespan, Uppsala University’s Centre for Women’s Mental Health, Uppsala University, Uppsala, Sweden

**Keywords:** Closeness, Separation, Newborn, Parent, Nepal

## Abstract

**Background:**

Closeness is crucial for the physical, emotional and social well-being of both parents and children. Although the benefits of closeness are proven for stable and unstable newborns, separation often remains standard, especially for small and/or sick newborns. We aimed to explore factors affecting the closeness and separation of parents and newborns in hospitals in Nepal.

**Methods:**

A qualitative design was used. Data were collected by individual interviews with 10 health care workers from labor rooms, postnatal wards or sick newborn care units/neonatal intensive care units in five referral hospitals in Nepal. Data were analysed using an inductive thematic approach.

**Results:**

Three main themes were generated: (1) *hospital resources*, (2) *in-hospital practices and attitudes*, and (3) *parental-newborn relationships and social factors*. Keeping the newborns spatially close to their mothers, offering a comfortable environment, and privacy were thought to enhance closeness, while heavy workload and lack of workforce hampered efforts to enhance closeness. Routines and rules separated parents and newborns, while actions and attitudes among health care workers strengthened closeness. Parental involvement and the influence of various social aspects, such as education, cultural beliefs and gender discrimination, were discussed.

**Conclusion:**

Although closeness was considered important, separation was common due to limited resources and existing rules and routines in the hospitals. Introducing small, low-cost changes in the wards, such as offering a comfortable place to sit, can help keep the parents close and lessen the workload for health care workers. To avoid separation, hospital rules and practices should be changed, and parents should be supported to take on the role of primary caregivers, with medical support from health care workers. There are traditions and cultural beliefs in society that hamper parental-infant closeness, and gender discrimination remains a problem.

**Supplementary Information:**

The online version contains supplementary material available at 10.1186/s12884-026-09387-5.

## Introduction

Although underfive mortality has decreased substantially over recent decades [1], neonatal mortality remains a global challenge. Among the 5 million children who died before their fifth birthday in 2020, almost half died in the neonatal period [2]. Reducing neonatal mortality is an urgent priority and has a particular target in Sustainable Development Goal 3 *Ensuring healthy lives and promoting well-being for all at all ages* [3]. The risk of death of a newborn is highest in the first 24 h after birth [2, 4], and it is crucial to improve the quality of care around birth to save lives [5–9]. This includes providing essential care for all newborns and special care for the most vulnerable, preterm, low birth weight, or sick newborns [10, 11]. Beyond survival, all children have the right to reach their full potential, according to the UN Convention on the Rights of the Child [12]. This is also incorporated in Sustainable Development Goal 3 [3], which states that all newborns should thrive and reach their full potential for health and life [13, 14]. Thus, quality postnatal newborn care must address immediate health concerns with evidence-based medical care, build parent-infant relationships, and establish behaviors that positively affect long-term infant development and health [15].

Parent-infant closeness is crucial for the physical, emotional, and social well-being of both parents and children [16]. It refers to physical and emotional closeness, incorporating bonding and attachment between parent and newborn, and evidence points out several advantages for the newborn. In addition to establishing the parent-infant relationship [17], the benefits of closeness range from avoiding hypothermia after birth [18, 19], increasing the rates of exclusive breastfeeding [20, 21], improving cognitive development during childhood [22–24], to increasing survival [7].

Skin-to-skin contact and breastfeeding might be the ultimate exercises of closeness and are the core components of kangaroo-mother-care (KMC), a recommended and life-saving method of care for preterm/low birth weight newborns [7, 25]. Even though the ‘natural place’ for a newborn is skin-to-skin with its mother and the benefits of skin-to-skin contact are proven for both stable and unstable newborns, separation remains standard in many hospitals, especially for small (premature or low birthweight) and/or sick newborns [26, 27]. This contrasts with international agreements such as the UN Convention on the Rights of the Child [12] and the 2030 Agenda for Sustainable Development [3], which underline the right to health and the right of children to be close to their parents.

In 2012, Flacking et al. [16] advocated that facilitating and inhibiting factors for closeness must be explored to implement appropriate strategies supporting the practice. Promoting closeness and avoiding separation might be particularly important to achieve survival and well-being in low- and middle-income countries, which face the highest burden of newborn mortality and morbidity. This study explores factors that affect the closeness and separation of parents and newborns in hospitals in Nepal.

## Methods

This study was part of the Nepal Perinatal Quality Improvement Project (NePeriQIP), a project aiming to improve quality of perinatal care through increased clinical skills and knowledge and to establish a structure for continuous quality improvement in hospitals in Nepal [28]. NePeriQIP was a collaboration between Ministry of Health and Population in Nepal and our research group at Uppsala university, Sweden, conducted April 2017- October 2018. It was rolled out in 12 public referral hospitals for maternal and newborn care in Nepal between 2017 and 2018. The primary outcome of the NePeriQIP trial was intrapartum-related mortality, and most of the studies from NePeriQIP was quantitative.

For the present study, a qualitative design was adopted, using individual interviews with health care workers (HCWs) to explore factors that may affect closeness and separation among parents and newborns in hospitals in Nepal.

### Study setting

The 12 hospitals included in NePeriQIP were selected by Ministry of Health and Population based on the criteria of having more than 1,000 deliveries a year and being public referral hospitals for maternal and newborn care. The interviews for this study were carried out at five of these hospitals which were chosen because of their different sizes, supporting between 1,000 and 11,000 deliveries per year. All hospitals provided vaginal and caesarian deliveries and had access to neonatal resuscitation services at birth. Healthy newborns were cared for in the postnatal wards. Sick, preterm, and/or low birth weight newborns were managed in sick newborn care units (SNCUs) or neonatal intensive care units (NICUs). The hospitals were located in rural areas mainly in flatlands and provided care to patients of diverse ethnicities, languages, and religions from surrounding areas. Two of the hospitals were found in the most disadvantaged regions in Nepal in terms of literacy, access to services, and life expectancy [29].

### Sampling and participants

A purposive sampling strategy was used to include HCWs from hospitals of different sizes. We selected one low-volume hospital (> 1,000 deliveries a year), two medium-volume hospitals (> 3,000 deliveries a year), and two high-volume hospitals (> 8,000 deliveries a year) from the hospitals participating in the NePeriQIP trial [28]. The criteria for inclusion were HCWs with varying experience and of different professions posted in the selected hospitals’ labor rooms, postnatal wards, or SNCUs/NICUs. The in-charge of the facilities and the data coordinators of NePeriQIP were asked to recommend potential participants who fulfilled the inclusion criteria. The HCWs were then informed about the study and invited to participate. In total, 10 HCWs were available during the data collection dates and consented to participate. Three of the respondents worked in the maternal ward (labor room, delivery room, and postnatal ward), one certified nurse-midwife (in a high-volume hospital), and two auxiliary nurse midwives (in medium- and small-volume hospitals). Five respondents were staff nurses working with sick, premature, and low birth weight newborns (two in medium-volume and three in large-volume hospitals). One respondent was a gynecologist (in a medium-volume hospital), and one was a pediatrician (in a large-volume hospital). The working experience among the respondents ranged from one month to 33 years.

### Data collection and analysis

Data were collected through individual interviews with HCWs during the intervention period of NePeriQIP on the topic of *closeness and separation between parents and newborns during the hospital stay.* The interviews were conducted in the hospitals by two public health professionals who were not part of the NePeriQIP intervention using a predeveloped question guide by the NePeriQIP-team (Supplementary material 1). Interviews with HCWs were performed after their working hours. No payment was provided for the HCWs, but transportation costs were covered. The interviews lasted from 10 to 30 min, were voice recorded, transcribed verbatim in Nepali by an independent team of trained research assistants, and then translated to English by a trained midwife researcher in Nepal. The translations were proof read by the interviewers.

Inductive thematic analysis, as outlined by Braun and Clarke [30], was used to analyse the interviews. For familiarization with the data, all interviews were read several times by the first author (OB). After this initial step, OB manually coded the entire dataset content. The initial list of codes was reviewed and refined together with second (AB) and last (LE) authors. Codes and extracts were then collated together in Microsoft Excel, and the codes were sorted into potential themes. This was a back-and-forth process undertaken by OB, AB, and LE, which resulted in the formation of themes and subthemes (Table 1). The list of themes and subthemes was then reviewed and refined at the level of coded extracts and in relation to the dataset. The final themes and subthemes were analysed, expressed in text, and discussed among all authors.

**Table 1:** Examples of coding and formation of subthemes and themes during the analysis

Extract	Code	Subtheme	Theme
Parents cannot touch and see their babies inside. They can only visit once, wearing a gown, and cannot touch their babies.	Parents not allowed to be close in NICU/SNCU	Rules in the hospital	In-hospital practices and attitudes
If we put the baby in skin-to-skin contact, the mother asks us to take the baby away from her. Some call their mothers and sisters, complain that their baby is placed on their chest, and request them to take the baby away… we don’t know why they say so. We don’t ask them.	Some mothers don’t want to have the baby on their chest	Parental involvement	Parental-newborn relationships and social factors

## Results

Our analysis generated three main themes: *hospital resources*,* in-hospital practices and attitudes*, and *parental-newborn relationships and social factors* (Table 2).

**Table 2:** The main themes and corresponding subthemes from interviews with health care workers about factors affecting parent-infant closeness and separation in hospitals in Nepal

Themes	Subthemes
Hospital resources	Spatial conditions
	Comfortable environment
	Privacy
	Workforce and workload
In-hospital practices and attitudes	Rules in the hospitals
	Routines in the hospitals
	Health care workers attitudes
	Health care workers’ actions beyond routines
Parental-newborn relationships and social factors	Parental involvement
	The role of cast, education, and poverty
	Gender discrimination

### Hospital resources

The health care workers highlighted the physical structure and organization of the facility as essential factors affecting the closeness between parents and newborns. Spatial proximity between mother and newborn was considered necessary to enable closeness; mothers should be kept close to SNCU/NICU or provided a bed inside to enhance bonding.

*“We must keep mothers and babies close to each other. The baby’s weight and breastfeeding get better if the baby gets proper care staying close to the mother. Staying together also helps in increasing the attachment of the mother and baby.”* (P9).

However, available infrastructure, space, and beds were often insufficient. For example, in several interviews, it was highlighted that the location of the wards was a problem. Newborns who were admitted were often separated from their mothers, and the maternal ward and the SNCU/NICU were far apart.

In some hospitals, kangaroo-mother care (KMC) could not be practiced inside the SNCU/NICU, as there was no room for mothers. In contrast, in one hospital, they had installed a particular corner where mothers of preterm babies could practice KMC. Likewise, even though the mothers could not stay with the newborns inside the wards, there was room inside the SNCU where they could breastfeed in one hospital. Little was said about how spatial conditions affected the father’s closeness with the newborn. However, one HCW mentioned that it was impossible to keep the fathers close to the mothers and newborns because of a lack of space.

Aside from the issue of spatial proximity, the quality of the physical environment as an enabling factor for closeness to occur was brought up as an area of improvement. This included aspects such as too high room temperature and the availability of beds or chairs that would allow the mother to sit comfortably when having her baby close. A comfortable place for the mothers was considered key to make them stay close to the newborn even when it was admitted to the SNCU/NICU for several days.

Another aspect of the physical environment that could be enhanced for closeness to happen was the aspect of privacy. It was mentioned, for example, that mothers may feel uncomfortable breastfeeding when there are many visitors at the ward or when, due to overcrowding of patients, two or even three babies were placed in the same bed. In some units, there were no separate nursing rooms or other adequate spaces for maintaining privacy. The lack of privacy also affected the birthing process.

*"Another thing we can do is not* *to make her [the mother] feel like she has committed a crime by giving birth to a baby*,* by making her birthing process comfortable. We can let her husband or other immediate caretaker stay with her during her birthing process. For that*,* the workload should be less. The room should be spacious*,* and privacy should be maintained by having one delivery in one room*,* instead of four to five.”* (P4).

Aside from the physical resources available at the hospital, HCWs stated that their ability to promote closeness diminished when they had too many or severely sick newborns in their care. The workload was described as too high and had not been for the students practicing in the wards in some hospitals; they would not manage at all. It was suggested that more human resources would allow for better promotion and support of evidence-based practices such as breastfeeding.

### In-hospital practices and attitudes

Several aspects relating to routines and rules, as well as health care workers’ actions and attitudes, were brought up as having potential impacts on closeness. There were, e.g., routines and rules that separated parents and newborns and hindered closeness as well as actions and attitudes among health care workers that strengthened closeness.

Mothers were generally allowed to be close to their babies to a greater extent than fathers, who were usually not allowed in the delivery room and sometimes not even in the maternity ward.

Pain after procedures such as vacuum extraction or episiotomy was sometimes referred to as an aspect that hindered the mother from being close to her baby. Additionally, after a caesarian section, the baby was not kept with the mother immediately; therefore, skin-to-skin contact or early breastfeeding was not initiated. However, if the mother could not be close to her baby, the father was often engaged and taught by the HCWs how to take care of his newborn. If the mother and her newborn were both healthy, the routine was to keep them together in the maternity ward, as it was considered necessary for attachment. If the baby was admitted to the SNCU/NICU, parents were not allowed to stay with their newborn. If a newborn was severely ill or due to the risk of infections, visiting the SNCU/NICU for hours and interactions between parents and the newborn could be restricted.

*“Parents cannot touch and see their babies inside [the ward]. They can only visit once*,* wearing a gown*,* and cannot touch their babies.”* (P5).

Other routines at the SNCU/NICU that also influenced the possibility of closeness negatively were, for example, when newborns were kept in radiant warmers, when nurses provided all the care for the newborns, including changing diapers and feeding, and when the newborns were not taken out of the SNCU/NICU to their mothers due to the risk of infections. Routines regarding KMC and breastfeeding were different between hospitals, even though they were considered important among all respondents. For example, in one hospital, all mothers of preterm babies were enabled to practice KMC, while in another, KMC was not practiced yet. In some places, the mothers were not allowed to breastfeed in the ward but in a room outside. Others were allowed to come and breastfeed in the ward but had to leave afterward.

Most HCWs highlighted that they routinely counsel and teach parents about breastfeeding, KMC, and how to take care of the newborn. In one hospital, they had group sessions before discharge, where all newborns were examined, and the HCWs provided counselling. However, it was mostly not the parents who accompanied the newborn during these sessions but rather relatives or neighbors, and essential information for the caretakers might have been lost.

The HCWs also spoke about their own opinions and attitudes regarding closeness. Several HCWs highlighted that providing treatment to the baby was easier when the newborn was together with the mother and that closeness was necessary for a good outcome. They expressed that skin-to-skin contact, early breastfeeding initiation, and counselling were vital in promoting closeness between the mother and her newborn. It was also mentioned that it was essential to enable the mothers to take care of their newborns in the hospital, doing everything from changing diapers to feeding. While fathers were mostly seen as visitors in the hospital, they should still be involved in the care of the newborn to improve the closeness.

Some HCWs did not believe that their own behavior affected closeness, that bonding between mother and newborn happens naturally, while others thought it played an important role.

*“Service providers’ behavior plays an enormous role*,* undoubtedly. While treatment*,* medicines*,* and proper care are essential*,* the service providers’ response to the visitors and parents is also crucial in building child bonding. For example*,* when the child is brought here for treatment*,* the parents are usually very nervous and tempered. Therefore*,* proper counselling from service providers is essential*,* and health care workers should always take responsibility for these things. We are here to treat patients*,* and taking responsibility seriously significantly impacts the overall condition of patients and visitors.* (P7).

In addition to routines, HCWs exemplified actions they took on their initiative to strengthen closeness. For example, one nurse said they had transferred beds from other wards to make it possible for mothers to stay close to their newborns admitted to the NCU. Failing to identify needs for extra attention and care was mentioned as something negative.

*“For example*,* yesterday*,* in the ward*,* someone told me that a baby had not been breastfeeding. I went there and taught the mother how to lift the baby and explained the proper positioning while breastfeeding the baby. The patient told me that she would have breastfed earlier if there were someone like me who explained everything so well.”* (P1).

HCWs tried to create an environment where mothers could be accompanied by their husbands or another family member to make it easier for them to care for the newborn. If a mother was unaccompanied in the hospital, one HCW said that they used to send a student to help her. HCWs also tried to involve the fathers or other family members more and taught them how to care for the mother and newborn.

One HCW said that they sometimes put the newborns to the breast even though milk was not produced or the baby could not suck effectively, just to keep the mother and newborn close. HCWs tried to ensure that the babies got to be with their mothers as much as possible, not keeping them for treatment or examinations more than necessary, and visiting hours were introduced for mothers to visit and care for their babies. However, one HCW mentioned that even though there were no formal rules regarding visiting hours, they still had to control the number of visitors to manage their duties. When mothers could not stay with their newborns in the SNCU/NICU, nurses sometimes took the baby to the mother to enable them to practice KMC.

### Parental-newborn relationship and social factors

The HCWs talked about the relationship between parents and newborns and the influence of various social factors. In the interviews, it was frequently mentioned that all parents loved their babies and showed loving behavior by trying to be close to their newborns, carry them and provide care for them. It was mentioned that parents liked getting detailed information about their newborn’s condition but that they did not want to interfere while HCWs provided care for them. Mothers were thought to be very happy when they could finally meet their babies after separation.

While mothers were often the ones who were physically closest to the baby, especially when breastfeeding, the fathers engaged in other ways. Loving fathers were described as being interested in the newborn’s condition, coming to visit often, and helping by bringing clothes or other materials. It was said that many fathers got time off from work to care for their baby and wife around birth, but in some communities, it was common for the father to work abroad, making him unavailable. In those cases, it was more common for the mother-in-law or sister-in-law to accompany the mother and help care for the newborn. Additionally, the existence of uncommitted or even abusive husbands was highlighted.

HCWs perceived that most parents complied with the counselling they received, as parents had the newborn’s best interest in mind. However, some mothers did not want to keep their newborns skin-to-skin.

*“If we put the baby in skin-to-skin contact*,* the mother asks us to take the baby away from her. Some call their mothers and sisters*,* complain that their baby is placed on their chest*,* and ask them to take the baby away… we don’t know why they say so. We don’t ask them.”* (P1).

Breastfeeding could also be an issue. After giving birth, some mothers did not want to breastfeed because they were in pain; others did not understand the benefits of breastfeeding and believed that breastmilk was not enough for the baby. However, one HCW also said that currently, mothers are aware of the importance of breastfeeding and that no mothers refuse to breastfeed their baby.

While most HCWs experienced that all parents cared for and loved their newborns equally, some thought the level of closeness varied among different groups. One HCW mentioned that cultural beliefs among the catchment population hindered closeness, which restricted older family members from caring for the mother and newborn.

*“There is a cultural belief not to touch mother or baby up to 11 days after birth. The older people do not provide any care; they don’t even touch. They even throw the baby’s clothes if they touch them; what care will they provide to the baby? There was a recent incident where the grandparents did not touch the baby while the mother had a postpartum hemorrhage. Because of that, even the baby got sick.”* (P1).

Another HCW perceived that the mothers from a specific community expected the nurses to care for the baby and did not do much themselves. She also thought their husbands did not care for their wives in the hospital.

Parents’ level of education and knowledge were also thought to impact closeness by some HCWs. It was mentioned that mothers with a higher level of knowledge tended to provide more care to the baby by themselves and that fathers and other family members acted more loving and caring if they were knowledgeable. On the other hand, it was said that illiterate people listened to HCWs and complied with their counselling. Poverty and stress were mentioned as other factors that negatively impacted closeness, and if the mother was unmarried.

Last, HCWs from two hospitals brought up discrimination against newborn girls as an issue relating to closeness. It was said that mothers did not usually discriminate by gender, but fathers and other family members did. If the parents already had several girls or if it was the first child, they were often disappointed, and the mother might be mistreated for having a girl. They were also thought to care less for baby girls, and sometimes necessary treatment was withheld from them.

*“There was a case where a mother gave birth to two preterm baby girls. Their condition at birth was not good. They could have been saved if the parents had taken them to a hospital that had a NICU. Although we counselled them about this*,* they refused to go there because the newborns were daughters. Eventually*,* both babies died here. The mother couldn’t do anything about it; it was the decision of her husband and his family members.”* (P3).

It was suggested that gender discrimination was only present among ignorant and uneducated fathers. If they were counselled or could be educated not to discriminate, they would take better care of their newborns, and closeness would improve.

## Discussion

This study explored factors affecting parent-infant closeness and separation in hospitals in Nepal. Physical and emotional closeness should be encouraged in hospital settings as part of quality care to increase survival, health, and well-being for all [16]. Nevertheless, separation remains standard in many settings [31]. In interviews with HCWs, we identified three main themes affecting closeness: (1) hospital resources, (2) in-hospital practices and attitudes, and (3) parental-newborn relationships and social factors.

### Hospital resources

The available resources of the hospitals were often insufficient to promote and maintain closeness. Spatial conditions and the physical environment separated parents and newborns, especially if the newborn was admitted to the SNCU/NICU. The maternity ward and SNCU/NICU were often far apart, and limited space was provided inside the SNCU/NICU for the parents, which is still the norm in many settings [32]. It is increasingly recognized that the architecture of the SNCU/NICU is essential to enhance closeness [16]. Nevertheless, the trend of changing the traditional open-bay design unit towards single-family rooms [33] is not feasible worldwide [34]. Another less costly way of facilitating parents’ presence in SNCU/NICU is providing comfortable chairs or beds at their newborn’s side [16, 35]. However, generally, there is a significant variation in the extent to which these kinds of facilities are offered in neonatal units [36, 37], and there was a shortage of them in our study as well. In addition, there were difficulties in maintaining privacy in the SNCU/NICU and maternity ward, a common problem in Nepal and other settings [37, 38]. This prevented mothers from keeping their newborns in skin-to-skin contact and from breastfeeding. Another factor related to hospital resources that was discussed to affect closeness was the lack of human resources, especially not having time to promote breastfeeding and skin-to-skin contact. In low- and middle-income countries such as Nepal, the nurse-baby ratio is low, making it challenging to provide quality care [39–42].

Organizational factors and architecture should be addressed to overcome the barriers of limited hospital resources. In India, the introduction of mother-newborn care units, with mothers present 24 × 7, showed an increased daily duration of skin-to-skin contact, higher breastfeeding rates, and lower mortality risk compared to conventional NICUs [32]. In addition, mothers substantially contributed to the care of the newborn in these units, including feeding, changing diapers, and monitoring babies for danger signs. This lessened the workload for HCWs and allowed them to teach the mothers healthy practices of neonatal care [32]. However, this requires reconstruction and is, of course, a matter of cost. A more feasible way to strengthen closeness might be to create space for the parents to stay near the SNCU/NICU and introduce minor, low-cost improvements inside the wards, such as having comfortable chairs at the bedsides and screens for privacy.

### In-hospital practices and attitudes

Rules and routines in the hospital separated parents and newborns in our study. Paternal involvement in pregnancy, delivery, and postpartum care has many benefits for both mothers and newborns. It has gained increased attention worldwide but is often overlooked in health care [43–45]. Likewise, in our study, fathers were mostly not seen as an integrated part of care and were restricted from being with their wives and newborns, although previous studies from Nepal have stressed their importance [46, 47].

If the newborn was admitted to the SNCU/NICU, separation was standard, and parents were often only allowed to spend minimal time with their newborns. This is common practice in many SNCU/NICUS around the world [26], despite evidence of the many advantages of keeping parents and newborns close [16, 22, 25].

The attitudes of HCWs are known to impact closeness [16, 48]. Most HCWs in our study expressed that they thought it was important to keep the mother and child together as much as possible and were aware of its physical and emotional advantages. Promoting skin-to-skin contact and breastfeeding were highlighted as critical components of supporting closeness. They knew that educating and informing parents is part of the quality of care [11]. Similar to a study by Feeley et al. [49], we found that HCWs undertook various actions to facilitate closeness but balanced this with medical needs and risks for the newborn. For example, HCWs feared neonatal infections and stopped parents from entering the SNCU/NICU. However, although hospital germs are potentially deadly and hygiene aspects such as handwashing remain essential, evidence that separation prevents infections is lacking [26].

HCWs are generally aware of the importance of keeping mothers and newborns in close contact, but hospital rules and practices need to change. The health benefits of closeness and the harms of separation should be acknowledged in hospital policies. It should be recognized that care and technology provided in SNCU/NICU do not require separation. Rather than being distant observers, parents should be involved as the primary caregivers of their newborns, with medical support from HCWs.

### Parental newborn relationship and social factors

HCWs thought that all parents loved their newborns and wanted to be close to them but that they accepted being separated and did not interfere with the care. While this can be expected in a hierarchical society where one obeys authorities, separation itself might also lead to less confidence in the parental role, and the stress of having a small and/or sick infant can lead to withdrawal physically and emotionally [48]. Husbands were absent during labor, which is common in Nepal depending on hospital rules, cultural beliefs, and traditions [46]. It was mentioned that husbands typically get parental leave to care for their wives and newborns in the hospital. Nevertheless, some fathers were working abroad, a known factor restricting men in Nepal from playing a part in pregnancy and childbirth [46].

Education and cultural beliefs also affect parents’ behavior and impact closeness; for example, the mother’s willingness to keep the newborn in skin-to-skin contact and to breastfeed. The practice of postpartum seclusion was mentioned, restricting husbands and family members from touching the newly delivered mother and child. This has also been reported earlier by Lewis et al. [46]. Discrimination against baby girls was sadly present in our study. Since 2015, Nepal has adopted its new constitution, which is committed to eliminating all forms of discrimination and achieving gender equality by 2030, as set out in the UN 2030 Agenda for Sustainable Development [3, 50]. Improvements have been made, but gender inequalities and discrimination remain in Nepal, now on 115 of 162 in the Human Development Report Gender Inequality Index [50].

Parental involvement in care needs to be encouraged in SNCU/NICU to strengthen their confidence as caregivers; a shift in their role from passive to active can enhance closeness. Public awareness of the benefits of breastfeeding and skin-to-skin care should be strengthened. Gender discrimination needs to be addressed further in Nepal.

### Strengths and limitations

Measures were taken to enhance trustworthiness of our findings, which typically involves the criteria credibility, transferability, dependability and confirmability.

#### Credability

There was a limited number of HCWs available for interviews during data collection. Thus, there is a risk that we did not reach what is sometimes referred to as saturation. However, saturation itself is complex and is debated for its controversies and contradictions [51, 52]. By interviewing additional HCWs, we might have gained more insight into factors affecting parental-newborn closeness and separation in hospitals. Nevertheless, we succeeded in interviewing HCWs from different professions, with varying work experience, and from different hospitals, which also reduces the risk of selection bias. Despite the limited number of participants, a variety of opinions and information was provided and all statements from the interviewees were given equal consideration.

The interviews were not conducted by any of the authors for linguistic and practical reasons. By using trained Nepalese data collectors who had no relation to the subjects or their workplace, and by guaranteeing the participants anonymity, we tried to limit the influence of social desirability bias. It is a limitation that we only explored the HCW perspective. It would be helpful and important for future research to incorporate the parents’ perspective. Provided the scarce literature on closeness from Nepal and similar settings, this article offers a description of factors affecting parent-infant closeness and separation.

#### Transferability

We have tried to describe the context and methods as thoroughly as possible to enable the reader to judge whether our findings are transferable to their own settings. Several of our findings have been described in others studies. For example, the finding that HCWs undertook various actions to facilitate closeness, but balanced them with medical needs and perceived risks for the newborn, is similar to a study by Feeley et al. [49] in Canada and Finland.

#### Dependability

We have reported about the context and processes undertaken within this study. We defined our sampling as purposive (hospitals) but the availability of health care workers during data collection created a limitation in the selection of participants. Still, a purposive sampling strategy is a common way of sampling in qualitative research, aiming to selecting a varying sample having valuable insights and representing various contexts.

#### Confirmability

We followed the step-by-step guide outlined by Brown and Clark [30] when analysing the data, but we acknowledge that there is always some level of interpretation done by the researcher. A rigorous back-and-forth process between several authors with different backgrounds was applied when coding and determining categories and themes. RG was familiar with the health-care settings where the research was conducted, while the other authors provided an outside perspective and all authors were involved in creating a consensus of the findings.

## Conclusion

We found several factors affecting parent-infant closeness and separation in hospitals in Nepal. Most elements were found to hinder closeness or increase separation due to limited hospital resources including low nurse-baby ratio and sub-optimal architecture of the hospitals, existing rules and routines in the hospital, traditions and cultural beliefs in the society, and gender discrimination. Simultaneously, existing attitudes among HCWs were found to promote closeness.

Organizational factors and architecture should be addressed to overcome the barriers of limited hospital resources, even if complex and costly. However, introducing small, low-cost changes in the wards can help keep the parents close and lessen the workload for HCWs. In addition, changing hospital rules and practices to keep parents and newborns together could be a significant achievement. Parents should be supported to take on the role of primary caregivers, with medical support from HCWs. Various traditions, cultural beliefs in society, and gender discrimination remain problems that Nepalese society needs to address.

## Supplementary Information

Supplementary Material 1.

## Data Availability

The datasets used and/or analysed during the current study are available from the corresponding author upon reasonable request.
