# Supplementary material for: Exploration of factors affecting parent-infant closeness and separation in hospitals in Nepal – a qualitative study
Source: BMC Pregnancy Childbirth. 2026 Jun 2;26:599. doi: 10.1186/s12884-026-09387-5 (PMC13231664; doi:10.1186/s12884-026-09387-5)
Supplement: Supplementary file 1 — Supplementary Material 1. [file 12884_2026_9387_MOESM1_ESM.docx]

**Questionnaire for interviews with Health Care Provider**

- How do you describe your experience with mothers and their immediate relatives regarding delivering services in labour room and SNCU/NICU?
- What do you feel about the current situation of labour room and SNCU/NICU?

Could you describe how you behaved with the postnatal women, their husbands and babies? To what extent do you feel you were able to address their wants and desires?

- In your opinion, what helps and what hinders the closeness between parent and baby in labour room and SNCU/NICU? (rules, number of caregivers, room structure, sense of responsibility of caregiver)
- We are grateful for your support by providing your suggestion and time regarding how effectively babies and their parents can be taken care of in hospitals of Nepal. Thank You!
